# Contribution of the Optical Coherence Tomography in Calcified Lesions

**DOI:** 10.31083/j.rcm2403093

**Published:** 2023-03-20

**Authors:** Nicolas Combaret, Nicolas Amabile, Benjamin Duband, Pascal Motreff, Géraud Souteyrand

**Affiliations:** ^1^Cardiology Department, Clermont-Ferrand University Hospital, CNRS, Université d’Auvergne, 63000 Clermont-Ferrand, France; ^2^Cardiology Department, Institut Mutualiste Montsouris, 75014 Paris, France

**Keywords:** coronary calcification, optical coherence tomography, optical frequency domain imaging - rotational atherectomy, orbital atherectomy, intravascular lithotripsy

## Abstract

Coronary artery calcification is a complex process found predominantly in the elderly population. Coronary angiography frequently lacks sensitivity to detect, evaluate and quantify these lesions. Yet calcified lesions are considered stable, it remains associated with a higher rate of peri procedural complications during percutaneous coronary intervention (PCI) including an increased risk of stent under expansion and struts mal apposition leading to poor clinical outcome. Intracoronary imaging (Intravascular Ultra Sound (IVUS) and Optical Coherence Tomography (OCT)) allows better calcified lesions identification, localization within the coronary artery wall (superficial or deep calcifications), quantification. This lesions characterization allows a better choice of dedicated plaque-preparation tools (modified balloons, rotational or orbital atherectomy, intravascular lithotripsy) that are crucial to achieve optimal PCI results. OCT could also assess the impact of these tools on the calcified plaque morphology (plaque fracture, burring effects…). An OCT-guided tailored PCI strategy for calcified lesions still requires validation by clinical studies which are currently underway.

## 1. Introduction

Coronary calcification formation is a process involving complex cellular regulatory mechanisms (osteogenic and osteoclastic). The main cause of atherosclerotic plaque calcification is the apoptosis of vascular smooth muscle cells leading to microcalcifications and the apoptosis of macrophages leading to larger calcium deposits [1, 2]. The microcalcifications will gradually coalesce to form larger calcium spots extending into the surrounding collagenous matrix [2]. Two types of coronary calcifications can be distinguished according to their location: (1) intimal calcifications in contact with the arterial lumen and (2) medial calcifications, which are deeper and alter arterial compliance. Both types of calcifications can be found in the same patient [1, 2].

The prevalence of coronary calcifications is more than 90% of men and 67% of women over 70 years of age [1] and several factors are known to favor their formation (advanced age, diabetes, chronic renal diseases, disorders of phospho-calcium metabolism) [1]. The overall ageing of the population is likely to lead to an increase in the prevalence of these calcified lesions. Percutaneous coronary interventions (PCI) of these lesions highly calcified coronary lesions are impacted by higher rates of short and long-term complications: increased 1-year rates of stent thrombosis (2.7%) and ischemic TLR (target lesion revascularization) (8.2%) [3, 4, 5].

It is therefore essential to identify these calcified coronary lesions to optimize their management. If calcium is angiographically visible that indicates an important load of calcium in the arterial wall. This could be correlated to an important risk of stent under expansion. In case of thinner calcium, coronary angiography significantly underestimates the incidence and severity of calcium load when compared to intravascular ultra sound (IVUS) and optical coherence tomography (OCT) [6]. Intracoronary imaging is not currently recommended as a routine procedure, although it has a place in certain situations such as analysis of the mechanisms of acute coronary syndromes or PCI of the left main coronary artery [7] but may have a role to play in the identification, evaluation and management of these calcified lesions [8].

OCT has an excellent spatial resolution that allows identification of calcified areas as small as 10 μm (compared to 200 μm with X ray conventional angiography) and may be the examination of choice for the analysis of calcified lesions. The sensitivity and specificity of OCT for calcified lesions identification are respectively 100% and 100% when compared to *ex vivo* histology [9]. This technique also makes it possible to assess the impact of different therapies on calcified lesions with specific signs that can only be visualized with the resolution of OCT [10, 11, 12, 13, 14].

This review paper aims to expose the potential benefits of OCT imaging in diagnosis, evaluation and therapeutic options management of coronary calcified lesions.

## 2. Calcified Lesions in OCT

### 2.1 Description of Calcified Lesions in OCT

**Fig. 1.**
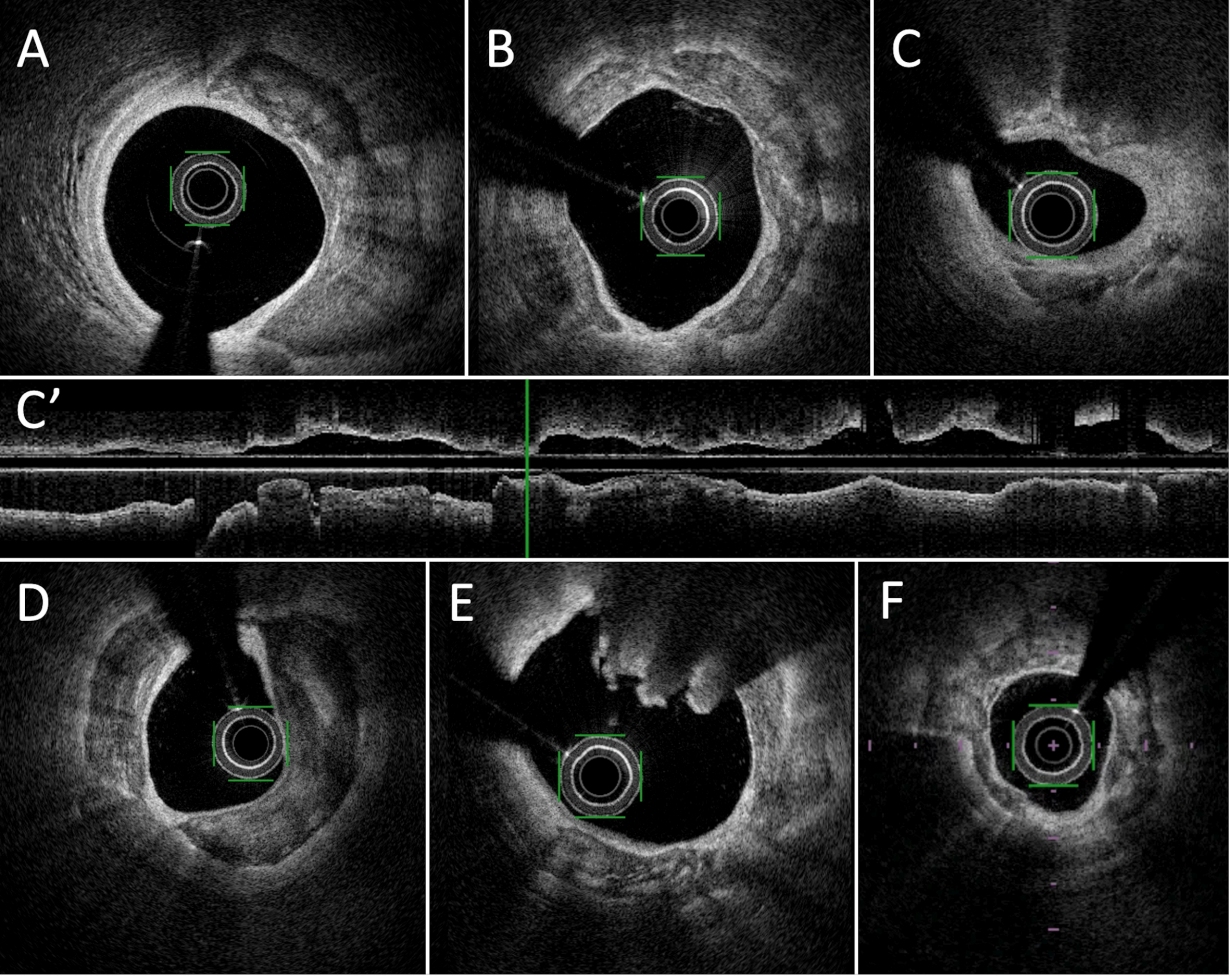
**Examples of calcified lesions in Optical Frequency Domain imaging (OFDI)**. (A) thick calcified lesion between 1 and 5 o’clock. (B) annular calcified plaque. (C) Deep calcified lesions between 11 and 1 o’clock and between 3 and 7 o’clock. (C’) long axis view of large calcified plaque in lower part of the panel. (D) massive deep and annular calcified lesions. (E) calcified nodule with superficial (luminal) calcification between 11 and 2 o’clock with cauliflower aspect and deep calcification at the opposite. (F) Annular calcification lesion with variable depth.

Calcium optical properties include limited backscattering and absorption of infrared photons emitted by OCT catheters [15]. Thus, the classic image of calcified lesion is an area of moderate hyposignal, with well-limited sharp borders and moderate signal attenuation, allowing analysis of the underlying structures [16]. Although this latter aspect may be modified in the presence of intraplaque lipids (mixed plaque with high infrared absorption) [17], it most permits OCT imaging, unlike IVUS, to measure calcification thickness and volume [18]. Given the spatial resolution of the technique, the threshold of detection of calcifications is estimated at 10–15 μm. Finally, longitudinal reconstruction view could be used to measure the calcified lesion length [19] (Fig. 1).

### 2.2 Calcified Lesions Characterization by OCT Imaging

Calcified lesion characterization by OCT analysis should include two types of information. First, their precise localization within the vascular wall needs to be assessed, leading to their classification as superficial (i.e., located between the intima and the media) or deep (located between the media and the adventitia). Second, the lesions have to be quantified.

The essential element of assessment is the radial extension of the calcification which can be done in continuous numerical form (absolute value in degrees, between 0 and 360°) or semi-quantitative (quadrants, between 0 and 4). This parameter allows distinction between eccentric (less than 180°/2 quadrants of extension) and concentric lesions (more than 270°/>3 quadrants). It should be noted that the latter is present in almost 20% of coronary lesions according to intra-coronary imaging analyses [6]. The other elements of assessment are the length, the longitudinal extension and the maximum thickness of the calcification. The combination of these parameters is used for the calculation of specific scores [20] or the measurement of calcific volume [18]. The latest generations of imaging software (with artificial intelligence techniques) automate this process for simpler and faster exploitation of these data [21, 22].

Noteworthy, the correlation between angiographic and intracoronary extension of calcifications is poor. A significant number of lesions (22 to 24%) without any suggestive image on angiography present significant calcification images (radial extension >90°) on intracoronary imaging. This confirms the inadequate performance of coronary angiography for this task. Nevertheless, there may be a relationship between angiographic severity on the one hand and longitudinal extension or maximum thickness of calcified areas on the other [23].

### 2.3 High Risk Calcified Lesions: Why Perform Intracoronary Imaging?

**Fig. 2.**
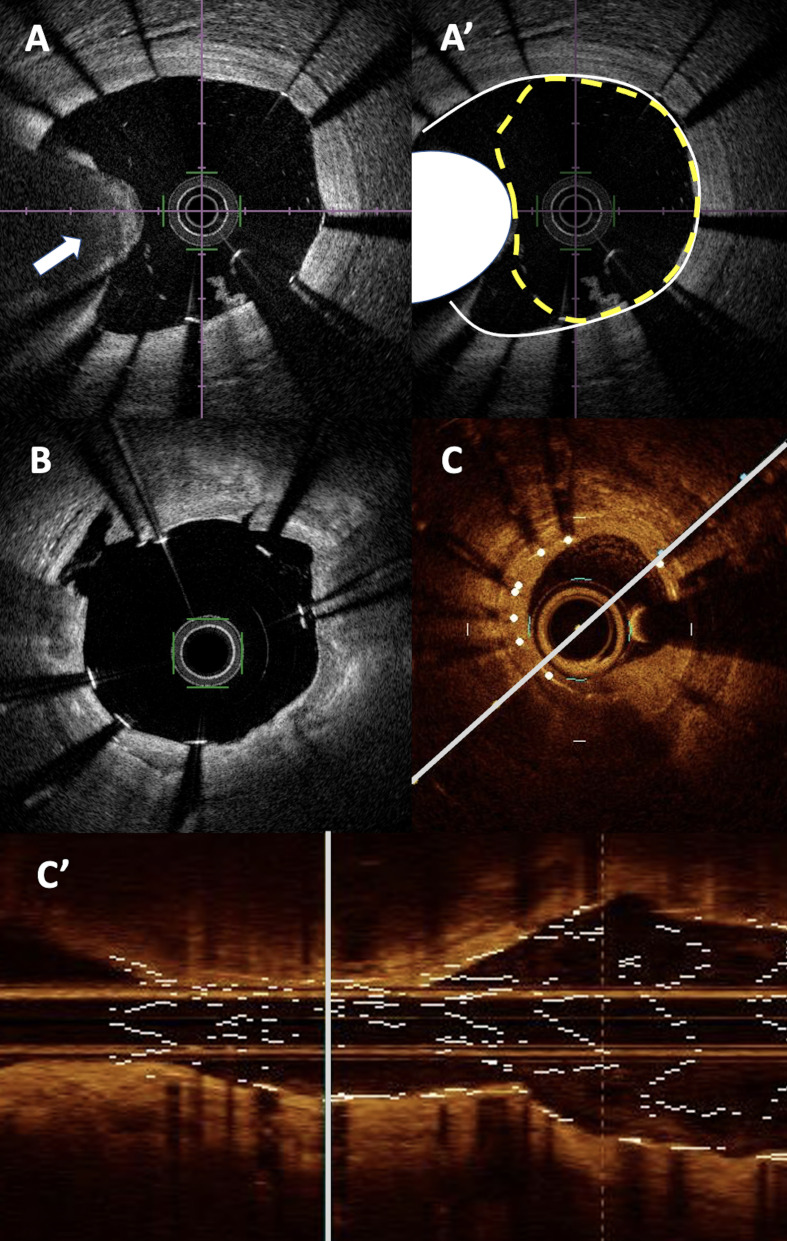
**PCI and calcified lesions in OCT**. (A,A’) massive calcified nodule between 8 and 10 o’clock (represented in white on A’ panel) leading to significant under-expansion of the stent (in yellow dot line) and mal apposition of the nearby struts. (B) Localized stent mal apposition (between 1 and 3 o’clock) after intravascular lithotripsy with visible plaque fracture at 1 o’clock and small intimal dissection at 10 o’ clock. (C) Moderate intra stent restenosis with significant stent under expansion next to an annular calcified plaque in short axis (C) and long axis view (C’). PCI, percutaneous coronary intervention; OCT, optical coherence tomography.

**Fig. 3.**
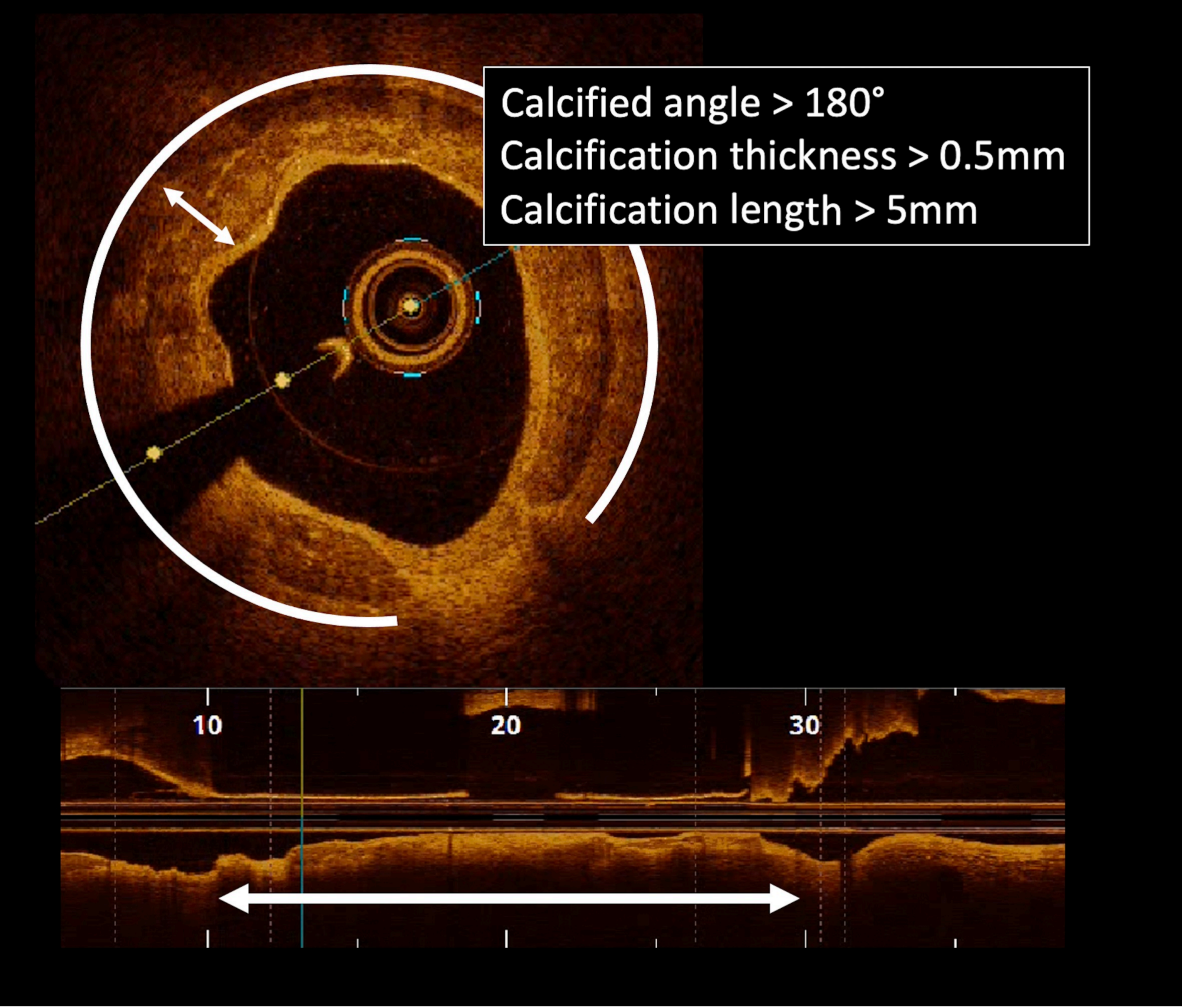
**Example of high risk calcified lesion with thickness >0.5 mm, length >5 mm and angular extension >180°**.

As explained previously, angiographic imaging may detect lesions with important thickness of calcium but in case of thinner calcified lesions, angiography alone can be taken in default [6] and intracoronary imaging and especially OCT can be an advantage. OCT imaging data could be used to identify calcified lesions associated with poor clinical outcomes, particularly due to under-expansion or stent malapposition [24, 25, 26] (Fig. 2). The elements of high risk calcified plaques are a calcification angle >180°, a maximum thickness >0.5 mm and a length >5 mm [14, 19, 20]. Ong *et al*. [27] established a calcific index defined by the product of the calcific arc and the length of the calcified plaque. Fujino *et al*. [20] proposed a score taking into account the above parameters (2 points for a calcific arc >180°, 1 point for a thickness >0.5 mm and 1 point for a length >5 mm). A high score is correlated with poor stent expansion (stent expansion <70% in 29.2% of cases with score of 4) [20] (Fig. 3). This may be explained by the difficulty, in the case of a high score, of obtaining a plaque fracture, which is a predictor of better stent expansion [28, 29].

Finally, some works have tried to define the best cutoff value of calcium thickness to predict calcium fracture observed in OCT: 0.67 mm when rotational atherectomy is used, 0.45 mm with cutting balloon and 0.24 mm with normal balloon [29, 30, 31].

### 2.4 The Calcified Nodule

Calcified nodules are specific calcified lesions characterized by the eccentric protrusion of a highly calcified atheromatous core through the fibrous layer of the plaque, creating a rupture of the latter [32]. Moreover, these lesions could also become unstable (accounting for 2 to 7 % of acute coronary syndromes) and be capped by fixed red thrombus [32]. These nodules are particularly at risk of per or post angioplasty complications [33]. OCT allows confirmation of the diagnosis of calcified nodules, with or without instability [34, 35]. These lesions are most often eccentric and present on the distal part of the left main coronary artery or the proximal part of the tortuous right coronary within a globally calcified artery [32, 36]. Their appearance is often smooth with microcalcifications on the surface.

**Fig. 4.**
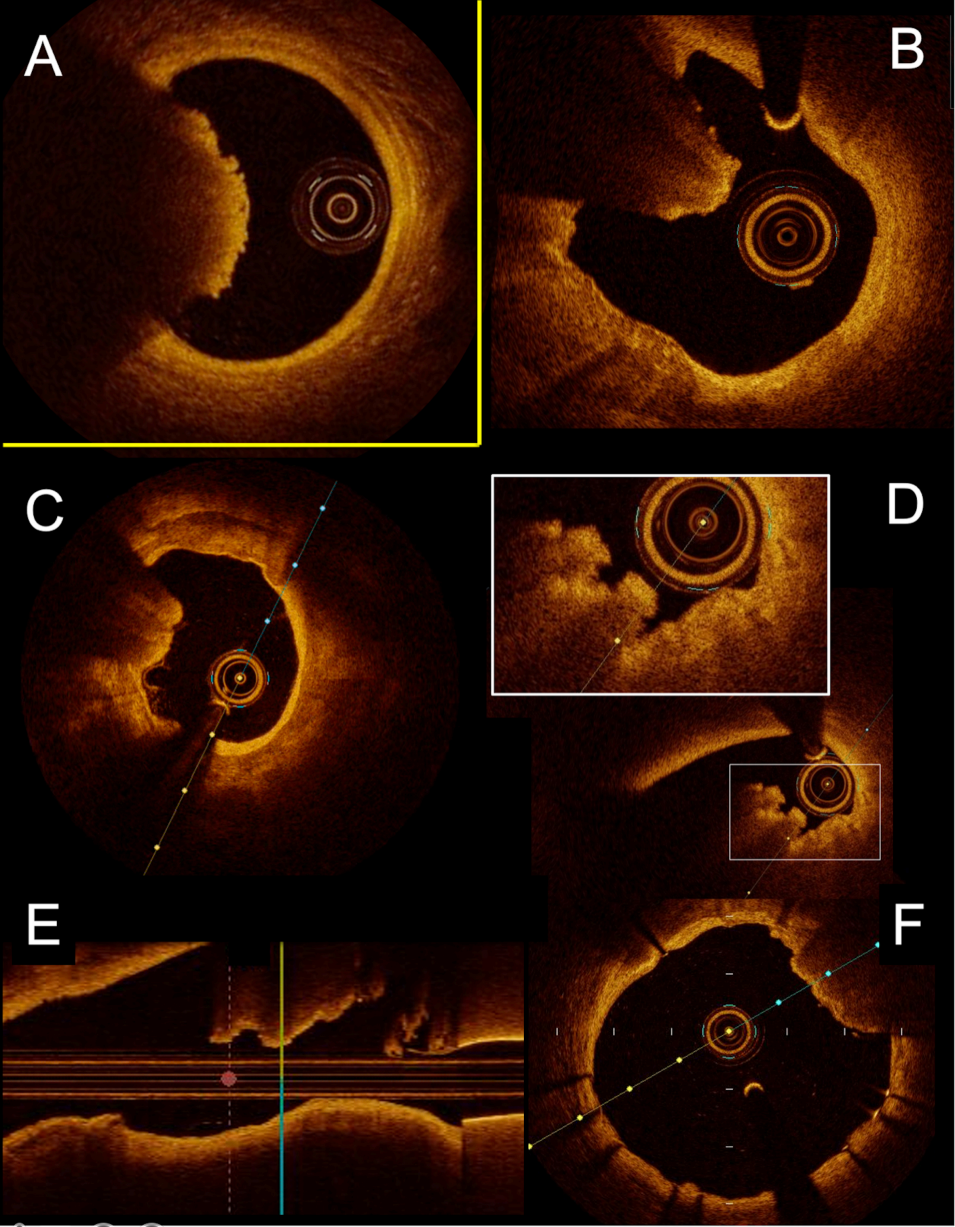
**Representative comparison between calcified nodule and red thrombus in OCT**. (A) typical aspect of red thrombus between 7 and 10 o’clock with significant attenuation of the signal. The normal aspect of the artery in 3 layers is clearly visible between 10 and 7 o’clock. (B) Calcified nodule with similar aspect in OCT (signal attenuation). The normal aspect of the wall is not present with an important calcified plaque between 6 and 11 o’clock. (C) Another example of calcified nodule at 9 o’clock and semicircular calcified plaque between 6 and 1 o’clock. (D) Another example of protrusing calcified lesion with lower signal attenuation and “cauliflower” aspect in short axis view. (E) Long axis view of the same lesion with abrupt transition between the calcified nodule and the normal aspect of the wall on the left side of the panel. (F) Post PCI imaging with moderate stent under expansion between 1 and 3 o’clock next to a calcified nodule. PCI, percutaneous coronary intervention; OCT, optical coherence tomography.

It is sometimes difficult, even on intracoronary imaging, to differentiate it from a true red thrombus [37] which often has a “cauliflower” appearance with multiple protrusions within the arterial lumen without a smooth underlying appearance (Fig. 4).

These calcified nodules, due to their intraluminal protrusive morphology, can cause stent under-expansion or eccentric deployment that is frequently difficult to correct. Morofuji *et al*. [38] demonstrate that the presence of calcified nodules in highly calcified lesions is associated with a poor clinical prognosis at 5 years. Furthermore, they can be the cause of true acute coronary syndromes and potentially sudden death by thrombus formation on the endoluminal calcified surface [32, 39].

## 3. OCT-Guided Management

Many tools dedicated to the preparation of calcified plaque are now available. The aim is to modify the mechanical properties of the plaque including its compliance. Ultimately, the purpose is to optimize stent implantation by optimizing sizing and reducing the risk of under-expansion and malapposition, which are correlated with a higher clinical event rate. These tools are: conventional non-compliant balloon, very high pressure non-compliant balloon, cutting and scoring balloons, laser, rotational atherectomy, orbital atherectomy and intravascular lithotripsy. Most of these different therapies have been shown to be effective in improving angioplasty outcomes in calcified lesions, but the best way to use them and their best indications remain debated [40]. Currently there are no randomized clinical trials or recommendations for the use of any of these tools based on intracoronary imaging data. Algorithms have been proposed with an emphasis on radial extension of calcium [40, 41].

De Maria *et al*. [40] proposed that in case of presence of calcium arc >180° and/or calcium length >5 mm and/or calcium thickness >500 microns specific plaque preparation by atherectomy (rotational or orbital) or endovascular lithotripsy should be used. In other cases, preparation of the lesion with a non-compliant balloon or cutting balloon is sufficient. If atherectomy or lithotripsy is necessary, analysis of the location of the calcium could guide the choice of one or other of the techniques: atherectomy in the case of intimal calcifications and lithotripsy in the case of deep calcifications, although to date no clinical study has validated this concept.

Sorini Dini *et al*. [42] proposed to establish a score taking into account the length of calcium >5 mm (1 pt), the thickness of the calcium >0.5 mm (1 pt) and the extent of the calcium arc (2 pts if 180–270° and 3 pts if arc >270°). For a score of 1 to 2, the use of non-compressing or high-pressure balloons is recommended as first-line treatment. For a score between 3 and 5, lithotripsy should be used as first line. In case lithotripsy balloon could not cross the lesion, atherectomy (rotational or orbital) is strongly recommended. Combined atherectomy and lithotripsy strategies are possible.

## 4. OCT Analysis after Treatment of Calcified Lesions

### 4.1 Impact of Different Therapies on Calcified Lesions

**Fig. 5.**
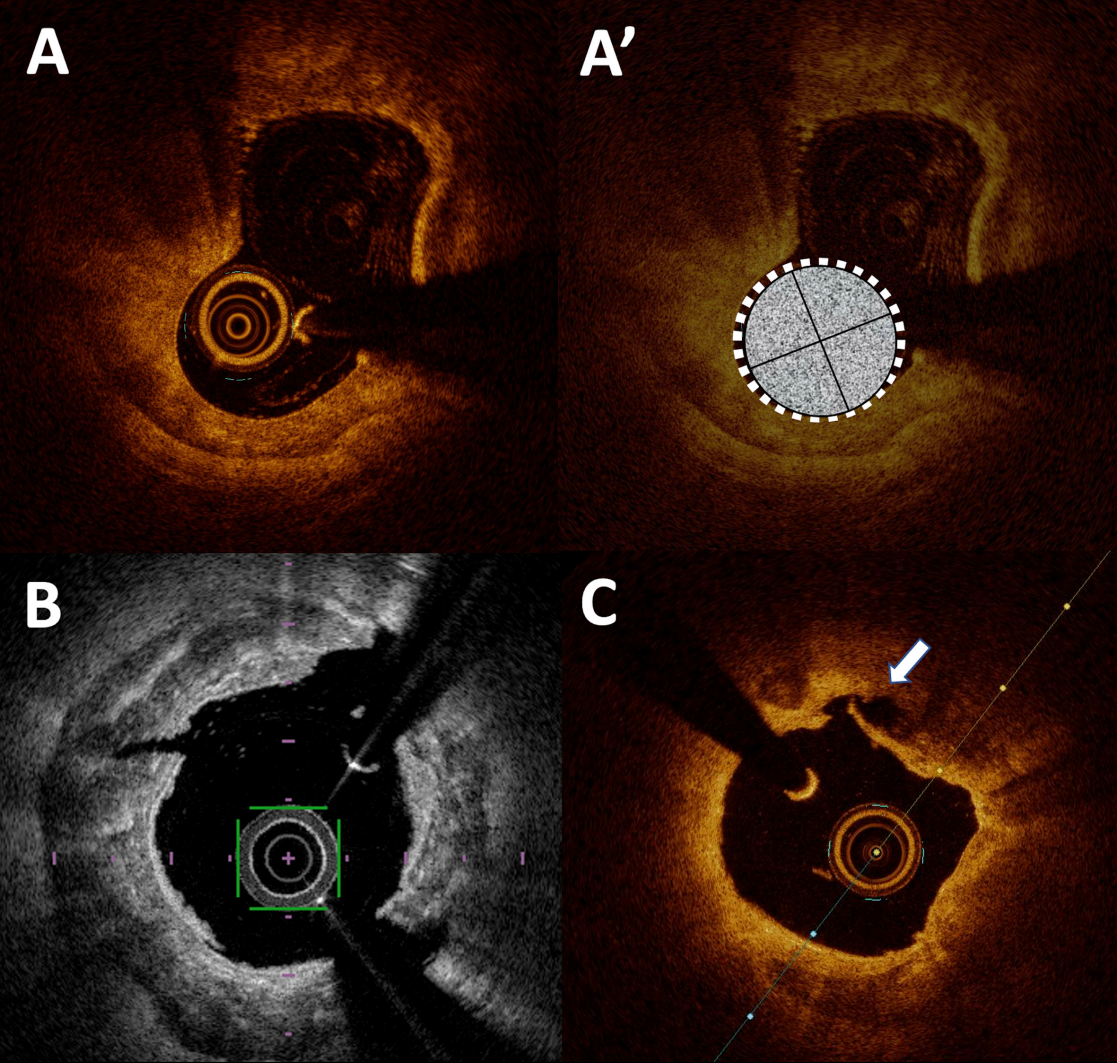
**Intra coronary imaging after calcified plaques preparation**. (A,A’) shows the effect of rotational atherectomy with a gutter aspect at the lower part of the artery. The burr is represented in (A’) by the grey circle. (B) Typical aspect of calcified plaque fractures at 1, 5 and 10 o’clock after intravascular lithotripsy. The fracture at 1 and 5 o’clock are associated with deep dissections. (C) Intimal dissection at 12 o’clock (white arrow) after calcified lesion preparation by orbital atherectomy.

Each calcified plaque preparation technique has clear OCT effects that are quite specific and reproducible (Fig. 5). The presence of these OCT signs could reflect the effectiveness of the technique used and should therefore be investigated.

### 4.2 Cutting Balloons

In the case of preparation with cutting or scoring balloons, OCT shows images of fissures or incisions in the surface of the plaque [41, 43]. The combination of cutting balloon predilatation after rotational atherectomy would result in more fractures within the calcified plaque than a combination of atherectomy and conventional balloon dilatation according to Amemiya *et al*. [44].

### 4.3 Atherectomy

Atherectomy (rotational or orbital atherectomy) creates abrasion of the calcified plaque by the burring effect that could combine with fracture lines within the calcified plate by the vibration effect [13]. Due to the anterograde operation of the burr, post rotational atherectomy lesions are preferentially located in the convex part of the angulations of the artery and appear as fracture lines sometimes as gutters. Orbital atherectomy will result in rounder, deeper and longer gutter images with a smooth appearance (“sanding”) within the calcified plaque [10, 13]. These two techniques lead to iatrogenic dissections which are more frequent and deeper within the calcified plaques which are richer in lipids [13].

Finally, although data are scarce, laser atherectomy is thought to cause fractures within the calcified plaque with multiple dissections sometimes relatively deep within the wall [45].

### 4.4 Intravascular lithotripsy

Intravascular lithotripsy induces disruption lines within the calcific structure. These fractures are multiple, relatively deep and visible longitudinally [11, 46].

A recent work compared the results of stent expansion according to the technique of preparation of the calcified plaque. No difference in terms of stent expansion was shown between rotational atherectomy, modified balloons (cutting or scoring balloons) or very high pressure balloons plaque preparation [47]. Some studies on this topic are currently in recruitment (NCT05301218, NCT05394649).

## 5. Conclusions

Optical coherence tomography is a major player in the management of calcified coronary lesions. Its spatial resolution allows confirmation of the calcified nature of a plaque, a feature often underestimated in angiography alone. Yet coronary calcifications are a source of complication during PCI (under-expansion, malapposition or stent fracture). OCT can be used to analyze the characteristics and the amount of the calcium within the plaque (radial extension, thickness and length). These parameters are crucial in order to identify high risk lesions for non-optimal PCI results. Although no study has yet been published, OCT appears as a promising supporting tool to guide the strategy and determine the most adequate device for plaque preparation (modified balloons, atherectomy or lithotripsy) depending on the characteristics of the calcified plaque. Finally, OCT identifies the effects of these tools on the calcified plaque and allows us to evaluate the effectiveness of plaque preparation for PCI optimization.
